# Hemipelvectomy for *Clostridium septicum* necrotizing soft tissue infection with extensive pelvic organ destruction: a case report

**DOI:** 10.1186/s12245-026-01276-0

**Published:** 2026-07-01

**Authors:** Ryosuke Omoto, Naoki Okada, Atsushi Watanabe, Taizo Nakanishi, Naoki Nakamoto, Satoshi Fujimi

**Affiliations:** Division of Trauma and Surgical Critical Care, Osaka General Medical Center, 3-1-56 Bandai-Higashi, Sumiyoshi-ku, Osaka, 558-8558 Japan

**Keywords:** *Clostridium septicum*, Necrotizing soft tissue infection, Hemipelvectomy, Colorectal cancer, Septic shock

## Abstract

**Background:**

*Clostridium septicum* is a highly virulent, gas-forming, anaerobic Gram-positive bacillus known to cause rapidly progressive necrotizing soft tissue infection that is often associated with gastrointestinal malignancy or immunocompromised states. Mortality remains extremely high, with many patients dying within the first 24 h of onset. Although aggressive surgical debridement is the cornerstone of treatment, cases requiring hemipelvectomy are exceedingly rare, and postoperative management of exposed pelvic organs has not been described in the relevant literature.

**Case presentation:**

A 73-year-old woman with ascending colon cancer developed sudden severe left leg pain and paralysis, and computed tomography revealed extensive gas from the left thigh to the pelvis. Upon arrival at our tertiary center, she was in septic shock with gas rapidly progressing to the high retroperitoneum. Emergent hip disarticulation was performed, and *Clostridium septicum* was isolated. Despite early source control, ongoing toxin-mediated necrosis extending beyond the limits of limb amputation necessitated left hemipelvectomy on Day 4. Following radical debridement, postoperative management became extremely challenging because multiple pelvic organs were exposed, with necrosis of the bladder and perforation of ureter, rectum and vaginal wall. Serial debridement and staged repairs were required over a prolonged course. Granulation of the pelvic wound gradually progressed, allowing split-thickness skin grafting on Day 88, after which wound healing stabilized. She was ultimately stable enough to return to the referring hospital on Day 187.

**Conclusions:**

This is an extremely rare case of *Clostridium septicum* necrotizing soft tissue infection requiring hemipelvectomy, demonstrating that aggressive and timely radical debridement can be life-saving, even in cases with massive pelvic involvement. Furthermore, we present the first documented example of stepwise management of exposed pelvic organs after hemipelvectomy performed for necrotizing soft tissue infection, providing valuable insights for clinicians facing similar catastrophic presentations.

## Background

Clostridial species are anaerobic, spore-forming, gas-producing Gram-positive bacilli known to cause fulminant necrotizing soft tissue infection (NSTI). They collectively account for approximately 10% of all NSTIs, and previous reports have shown that *Clostridium septicum (C. septicum)* represents only 1–1.3% of all clostridial infections, making it an exceedingly rare cause of NSTI [1, 2]. Unlike *Clostridium perfringens*, which typically develops after traumatic injury or contaminated wounds, *C. septicum* infection most commonly arises spontaneously in association with malignancy, particularly colorectal or hematologic cancer, as well as immunosuppression or metabolic disorders such as diabetes [3–5]. The entry of the organism into these settings is thought to be a defect in the mucosal lining of the bowel wall caused by tumor, chemotherapy, ischemia, and perforation [6]. 

A key pathogenic feature of *C. septicum* is the action of its α-toxin. Unlike the phospholipase C α-toxin produced by *Clostridium perfringens*, the α-toxin of *C. septicum* is an aerolysin-like pore-forming cytolysin that induces rapid cell necrosis and endothelial injury, leading to microvascular thrombosis and extensive tissue devascularization [7, 8]. These mechanisms result in rapid tissue destruction and systemic toxicity. Because of its ability to proliferate in relatively aerobic environments and disseminate hematogenously, *C. septicum* infection progresses far more rapidly than other clostridial infections, with reported mortality rates ranging from 48% to 67%, and many deaths occurring within the first 24 h of onset [3, 5, 9].

To date, there are no documented cases of NSTI requiring hemipelvectomy with extensive destruction of multiple pelvic organs, and no established strategy exists for managing the exposed pelvic cavity that results from such radical debridement. This report describes an extremely rare case of *C. septicum* NSTI associated with ascending colon cancer that necessitated early hemipelvectomy and resulted in severe multi-organ pelvic involvement, and it outlines the complex postoperative management that ultimately led to the patient’s survival.

## Case presentation

**Fig. 1:**
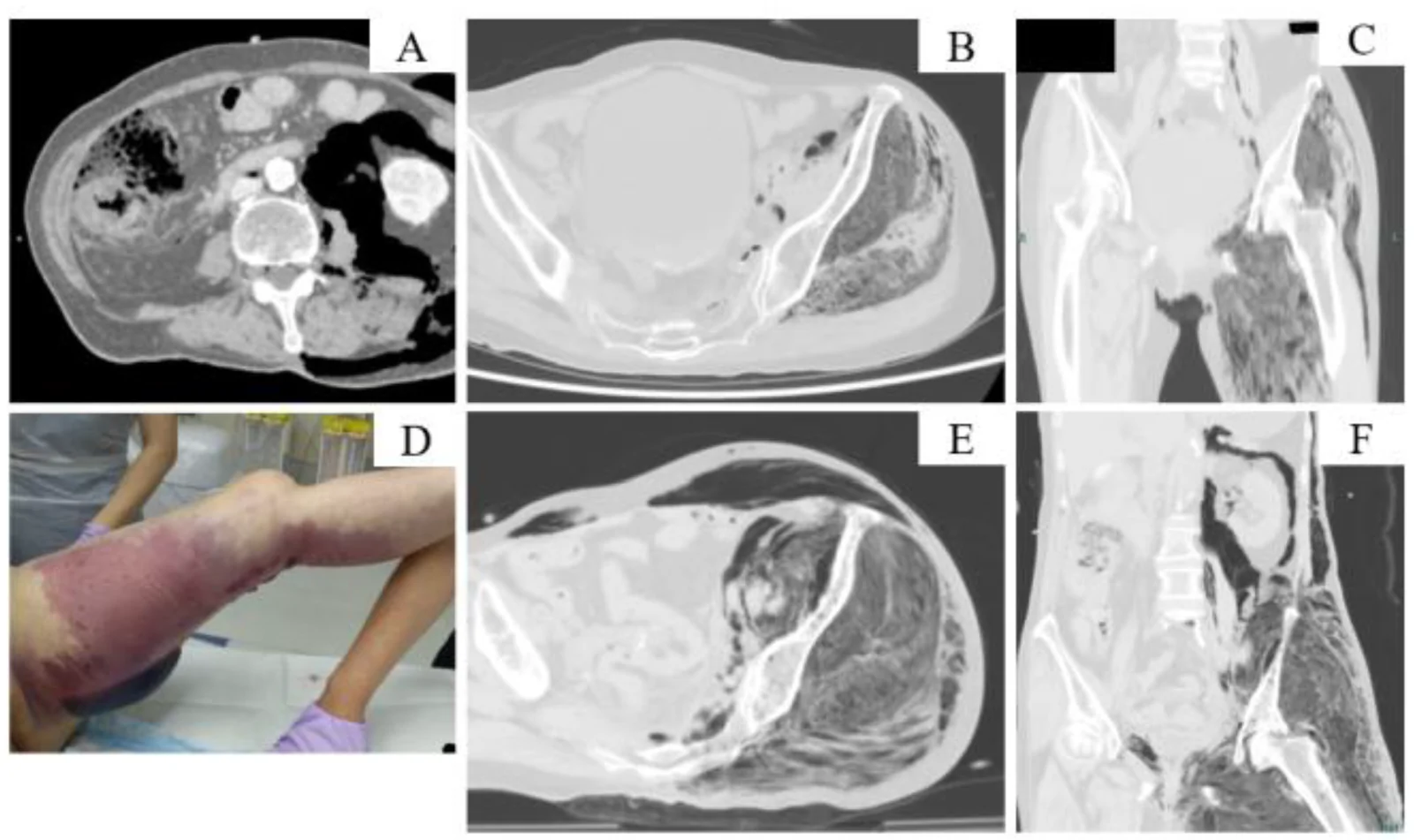
Clinical and radiological findings at presentation. (**A**) CT performed at the referring hospital shows ascending colon cancer with penetration toward the mesentery. (**B**, **C**) CT images obtained at the referring hospital demonstrate extensive gas formation confined to the left thigh and pelvic cavity. (**D**) Clinical photograph of the left thigh on arrival at our hospital shows marked skin discoloration with a violaceous appearance and bullae formation. (**E**, **F**) CT images obtained at our hospital demonstrate rapid progression of gas into the retroperitoneum surrounding the left kidney

**Fig. 2:**
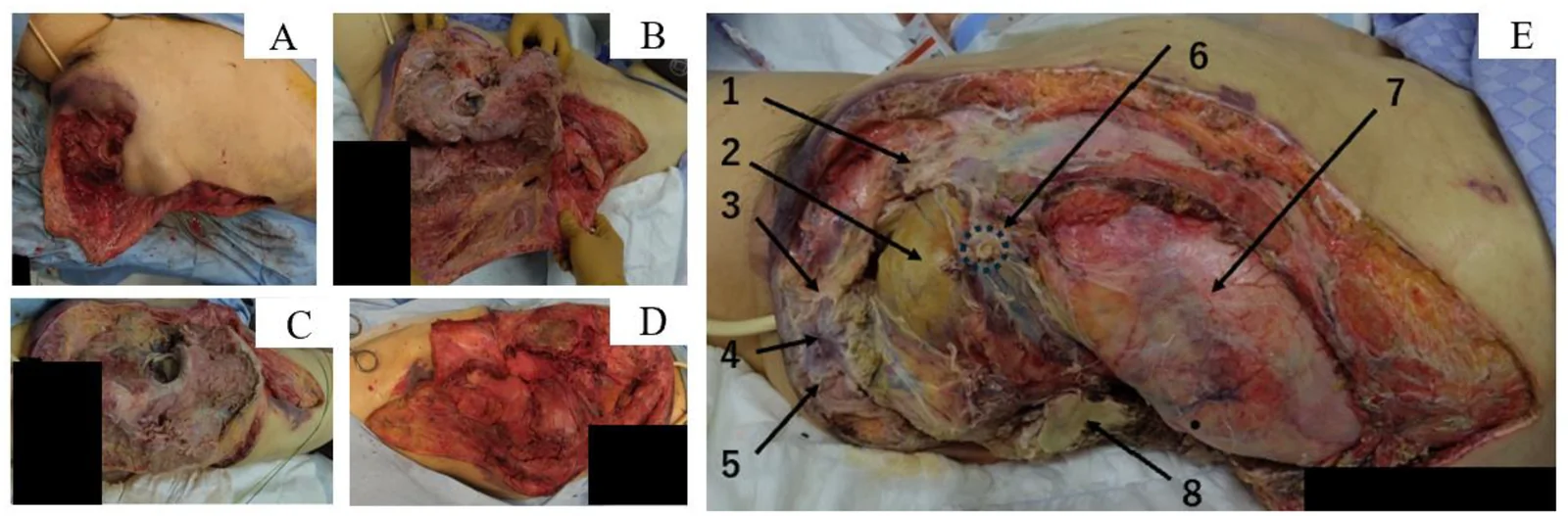
Intraoperative findings during staged debridement and hemipelvectomy. (**A**, **B**) Operative photographs on Day 1 after left hip disarticulation show residual discolored muscles with poor viability. (**C**) Operative photograph on Day 4 before hemipelvectomy shows residual devitalized muscles surrounding the pelvis. (**D**) Operative photograph after left hemipelvectomy on Day 4 demonstrates complete debridement of all nonviable muscles. (**E**) Operative photograph obtained on day 7 demonstrating the pelvic anatomy after hemipelvectomy: (1) pubic symphysis, (2) necrotic bladder wall, (3) urethra, (4) vagina, (5) rectum, (6) stump of the common iliac artery, (7) peritoneum, and (8) sacroiliac joint

A 73-year-old woman was admitted to a hospital for planned surgery for ascending colon cancer (cT3N1M0, cStage IIIb) with mesenteric penetration (Fig. 1A). Her past medical history was significant for hypertension and well-controlled diabetes mellitus (HbA1c: 6.7%). She suddenly developed pain and paralysis of the left lower limb, and computed tomography (CT) revealed extensive gas formation from the left thigh to the pelvic cavity, prompting transfer to our hospital (Fig. 1B.C). She was transported to our hospital approximately 5 h later. On arrival, her vital signs were as follows: Glasgow Coma Scale, E4V5M6; blood pressure, 90/50 mmHg; heart rate, 93 bpm; respiratory rate, 30 breaths/min; SpO_2_, 100% on room air, and body temperature 34 °C, indicating septic shock. An arterial blood gas analysis showed the following: pH, 7.23; base excess, − 15.6; and lactate, 9.2 mmol/L. Laboratory tests revealed the following: white blood cell count, 6,100/µL; C-reactive protein, 16.8 mg/dL, and procalcitonin 59.1 ng/mL. The Sequential Organ Failure Assessment (SOFA) and Acute Physiology and Chronic Health Evaluation II (APACHE II) scores were 8 and 21, respectively. The skin of the left thigh was markedly discolored with bullae formation and crepitus (Fig. 1D). Repeat CT at our hospital demonstrated rapid progression of gas, now extending to the left perirenal retroperitoneal space (Fig. 1E.F). Emergency left hip disarticulation and debridement of the left flank soft tissue were performed, and *C. septicum* was isolated from both blood and wound cultures (Fig. 2A.B). An ileostomy was created on Day 3. The left hemipelvis was exposed, but the ilium and ischium had become sequestrated, and residual pelvic muscles, including the iliacus and psoas, were necrotic. Persistent progression of necrosis involving the pelvis despite prior hip disarticulation prompted left hemipelvectomy on Day 4 for definitive source control (Fig. 2C.D). The pelvis was transected anteriorly at the pubic tubercle and posteriorly at the sacroiliac joint, and the common iliac artery was ligated. The bladder, urethra, vagina, and rectum were left exposed, as shown in Fig. 2E. Vasopressors were successfully discontinued on Day 6. A tracheostomy was performed on Day 11, and she was weaned off mechanical ventilation on Day 18.

### Soft-tissue management

**Fig. 3:**
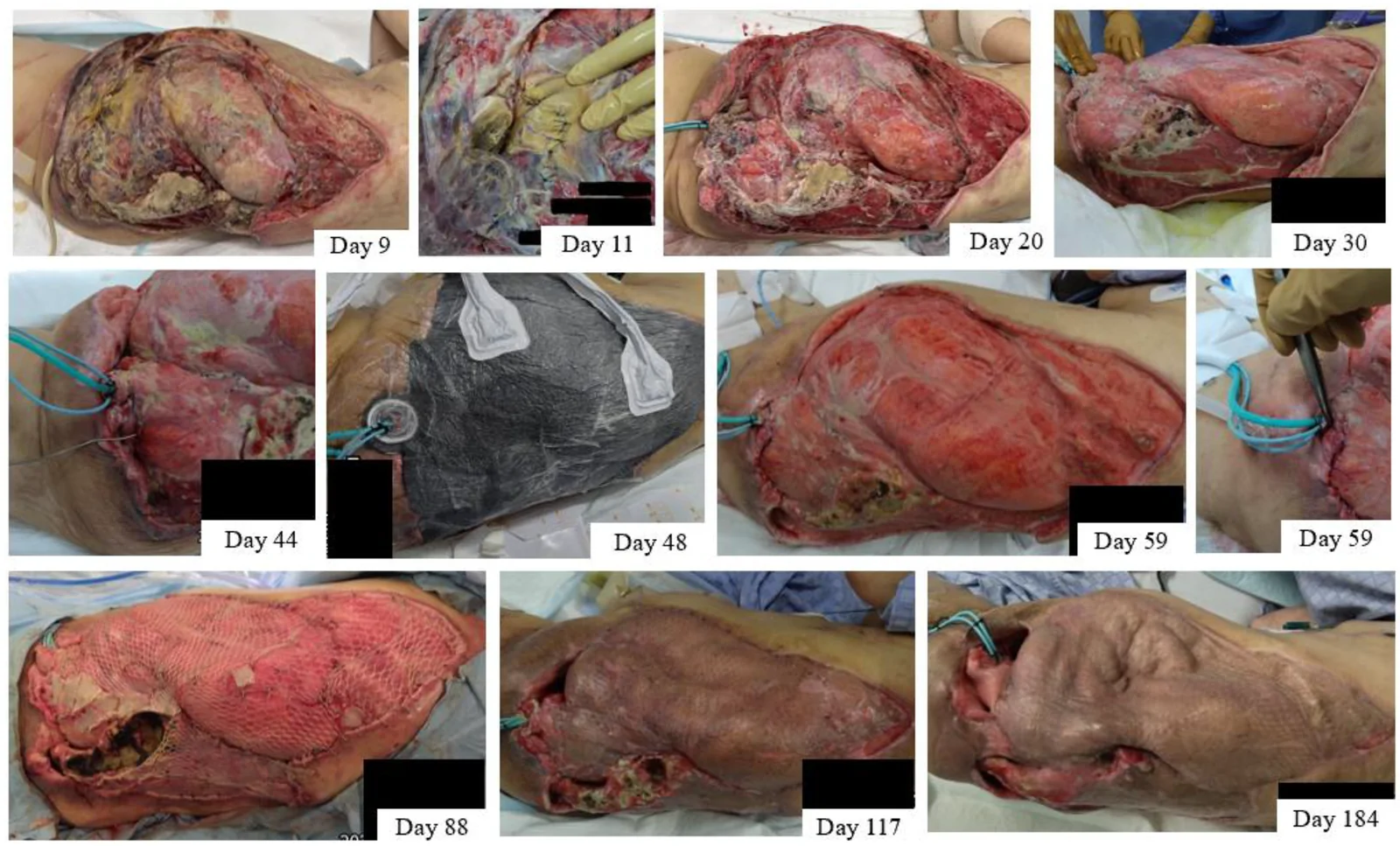
Sequential changes in the soft-tissue wound following hemipelvectomy. Serial photographs show the progression of the soft-tissue wound after hemipelvectomy. During negative pressure wound therapy (NPWT), fecal and urinary leakage occurred, and a wound-edge protection device (Lap-Protector) was used to isolate the pelvic organs and allow continuation of NPWT. Gradual granulation developed, and split-thickness skin grafting was performed on Day 88 with good graft take

Serial debridement was performed as needed, and intra-soft tissue antibiotic perfusion (iSAP) was initiated on Day 5, with gentamicin administered at 180 mg/Day and SI-mesh used as the contact layer. On Day 19, wound management was transitioned to a RENASYS™ NPWT system, using Sorbact^®^ as the contact layer. However, during the clinical course, wound contamination repeatedly occurred due to fecal contamination and urinary leakage. Therefore, both the urethral and rectal openings were isolated using a wound-edge protection device (Lap-Protector), enabling continued NPWT while minimizing further contamination of the wound bed. Granulation gradually progressed, enabling split-thickness skin grafting on Day 88. The graft demonstrated good viability and was mostly epithelialized, with the exception of the perirectal region and the space of Retzius (Fig. 3).

### Gastrointestinal management

An exposed rectum (Rb) perforation was detected on Day 17 and repaired; however, complete closure was difficult, and on Day 23, resection of the rectum (Rb) was performed. On Day 44, leakage from the rectal stump was observed again, resulting in exteriorization. Although recurrent stool contamination persisted, resection of the rectum (Ra) was performed on Day 71. After epithelialization of the skin graft, the rectal stump eventually dehisced and became difficult to close; however, it could be managed by applying gauze as a new anal outlet.

### Urinary tract management

The left side of the bladder was completely necrotic; therefore, partial cystectomy and bladder repair were performed on Day 11. On Day 17, left ureteral injury was identified, and bilateral SJ catheters were inserted. Subsequently, the bladder wall repeatedly dehisced and was repaired, eventually resulting in a markedly reduced bladder measuring approximately 2 × 2 cm.

### Gynecologic organ management

A vaginal wall injury was detected on Day 16, which was managed with simple closure.

Although she developed multiple complications, including persistent candidemia and acute kidney injury requiring renal replacement therapy (RRT), she was discharged from the ICU on Day 131 and weaned off RRT on Day 167. The patient ultimately survived with staged organ management and returned to the referring hospital on Day 187. At the time of transfer, her activities of daily living were limited to supported sitting. During her hospitalization, her tumor marker levels remained stable, and imaging showed no evidence of distant metastasis.

### Discussion and conclusions

Spontaneous *C. septicum* infections may spread through two recognized pathways following translocation across the compromised colonic mucosa: direct extension along the retroperitoneal or fascial planes, and hematogenous dissemination, resulting in metastatic myonecrosis involving multiple, noncontiguous muscle groups [3, 4, 10]. Although gas formation in the ascending colon and the left lower extremity appeared radiologically discontinuous in the present case, the infection remained confined to a single anatomical axis (from the left thigh to the pelvis and retroperitoneum) without involvement of distant muscle groups. Hematogenous metastatic myonecrosis typically exhibits multifocal involvement due to bloodstream seeding, which was not observed [3, 5, 10]. Furthermore, prior reports have noted that *C. septicum* can propagate rapidly through retroperitoneal fascial planes in a manner that may appear noncontiguous on imaging [11]. Taken together, these findings indicate that the infection in this patient most likely represented rapid direct extension rather than true hematogenous dissemination.

In the present case, infection progressed beyond the limits typically amenable to limb amputation, with ongoing necrosis of the iliac, psoas, and pelvic soft tissues despite hip disarticulation, ultimately necessitating early hemipelvectomy to achieve definitive source control. In contrast to most reported cases of hemipelvectomy, radical debridement in this patient resulted in simultaneous exposure and partial necrosis of multiple intrapelvic organs, including the bladder, urethra, rectum, ureter, and vaginal wall. To our knowledge, such extensive pelvic visceral involvement following hemipelvectomy for NSTI has not been previously described. Importantly, this case demonstrates that even extraordinarily advanced infections can be survivable when timely and appropriately aggressive debridement is combined with meticulous, staged postoperative management of exposed pelvic organs.

Traumatic hemipelvectomy provides an important point of comparison for understanding the unique challenges encountered in this case. To the best of our knowledge, only eight cases of traumatic hemipelvectomy have been reported in the literature. Among these, two cases required prolonged soft-tissue management because of severe wound contamination and subsequent soft-tissue infection: Zheng et al. described a patient who was dragged into a cement mixer and sustained an associated anorectal injury, requiring approximately two months of soft-tissue management, whereas Temizsoy et al. reported a case following a traffic accident with bladder and rectal injuries that was complicated by flap necrosis and infection, necessitating approximately six weeks of additional wound care [12, 13]. In addition, a previously reported case series of traumatic hemipelvectomy described exposure of pelvic organs, such as the rectum, bladder, and vagina; however, these organs remained well perfused and did not develop ischemic necrosis or perforation, and management primarily focused on soft-tissue control and wound coverage rather than visceral reconstruction [14]. Notably, even in these severely contaminated and infected traumatic cases, complex protection or reconstruction of intrapelvic viscera was not required. These observations indicate that the unprecedented difficulty in managing pelvic organs in the present case was not inherent to the hemipelvectomy procedure itself, but rather reflected infection-related devascularization driven by the fulminant pathophysiology of *C. septicum*, including potent α-toxin-mediated cytotoxicity, microvascular thrombosis, and rapid tissue ischemia [7, 8].

In our patient, exposure of the pelvic viscera required specific modifications of NPWT as part of soft-tissue management. Urine was repeatedly aspirated into the NPWT system because of the necrotic bladder wall, and fecal contamination occurred despite diversion procedures. To mitigate these problems, we placed a wound-edge protection device (Lap-Protector) around the exposed viscera and combined it with a non-adherent contact layer beneath the foam dressing, thereby physically isolating the bladder and rectum from the negative-pressure field and reducing contamination of the wound bed. This strategy conceptually parallels techniques used in the management of enteroatmospheric fistulas, in which NPWT is applied together with isolating adapters or silo-type devices to separate effluent from the surrounding open abdomen while allowing wound conditioning [15]. Saverio et al. have emphasized that the core strategy is rigorous isolation in the management of enteroatmospheric fistulas, and they have also emphasized that no universal solution exists and a degree of situation-specific improvisation is often required because each fistula and wound is highly individualized [16]. Although our approach may not be universally applicable, it nevertheless illustrates one practical example of how such challenging wounds can be successfully managed.

Although survival was achieved, functional recovery remained limited at the time of transfer, and long-term rehabilitation and oncologic outcomes were unavailable after transfer to another institution. This case demonstrates that even necrotizing soft-tissue infection with extensive pelvic involvement can be survivable with prompt radical debridement. Moreover, it provides the first case-based description of the postoperative management of exposed pelvic organs following hemipelvectomy for *C. septicum* infection.

## Data Availability

Data sharing is not applicable to this article as no datasets were generated or analyzed during the current study.

## Abbreviations

NSTI: Necrotizing soft tissue infection
C. septicum: Clostridium septicum
iSAP: intra-soft tissue antibiotics perfusion
RRT: Renal replacement therapy
